# Melatonin Improves Lipid Homeostasis, Mitochondrial Biogenesis, and Antioxidant Defenses in the Liver of Prediabetic Rats

**DOI:** 10.3390/ijms26104652

**Published:** 2025-05-13

**Authors:** Milena Cremer de Souza, Maria Luisa Gonçalves Agneis, Karoliny Alves das Neves, Matheus Ribas de Almeida, Geórgia da Silva Feltran, Ellen Mayara Souza Cruz, João Paulo Ferreira Schoffen, Luiz Gustavo de Almeida Chuffa, Fábio Rodrigues Ferreira Seiva

**Affiliations:** 1 Department of Parasitology, Immunology and General Pathology, State University of Londrina (UEL), Londrina 86057-970, Paraná, Brazil; 2 Department of Chemistry and Biochemistry, São Paulo State University (UNESP), Botucatu 18618-693, São Paulo, Brazil; 3 Center of Biological Sciences, State University of Northern Paraná (UENP), Bandeirantes 86360-000, Paraná, Brazil

**Keywords:** melatonin, prediabetes, mitochondrial dynamics, energy metabolism, oxidative stress

## Abstract

Type 2 diabetes mellitus represents a major global health burden and is often preceded by a prediabetic state characterized by insulin resistance and metabolic dysfunction. Mitochondrial alterations, oxidative stress, and disturbances in lipid metabolism are central to the prediabetes pathophysiology. Melatonin, a pleiotropic indolamine, is known to regulate metabolic and mitochondrial processes; however, its therapeutic potential in prediabetes remains poorly understood. This study investigated the effects of melatonin on energy metabolism, oxidative stress, and mitochondrial function in a rat model of prediabetes induced by chronic sucrose intake and low-dose streptozotocin administration. Following prediabetes induction, animals were treated with melatonin (20 mg/kg) for four weeks. Biochemical analyses were conducted to evaluate glucose and lipid metabolism, and mitochondrial function was assessed via gene expression, enzymatic activity, and oxidative stress markers. Additionally, hepatic mitochondrial dynamics were examined by quantifying key regulators genes associated with biogenesis, fusion, and fission. Prediabetic animals exhibited dyslipidemia, hepatic lipid accumulation, increased fat depots, and impaired glucose metabolism. Melatonin significantly reduced serum glucose, triglycerides, and total cholesterol levels, while enhancing the hepatic high-density lipoprotein content. It also stimulated β-oxidation by upregulating hydroxyacyl-CoA dehydrogenase and citrate synthase activity. Mitochondrial dysfunction in prediabetic animals was evidenced by the reduced expression of peroxisome proliferator-activated receptor gamma coactivator-1 alpha and mitochondrial transcription factor A, both of which were markedly upregulated by melatonin. The indolamine also modulated mithocondrial dynamics by regulating fusion and fission markers, including mitosuin 1 and 2, optic atrophy protein, and dynamin-related protein. Additionally, melatonin mitigated oxidative stress by enhancing the activity of superoxide dismutase and catalase while reducing lipid peroxidation. These findings highlight melatonin’s protective role in prediabetes by improving lipid and energy metabolism, alleviating oxidative stress, and restoring mitochondrial homeostasis. This study provides novel insights into the therapeutic potential of melatonin in addressing metabolic disorders, particularly in mitigating mitochondrial dysfunction associated with prediabetes.

## 1. Introduction

Diabetes mellitus (DM) is a chronic non-communicable disease (CNCD) characterized by persistent hyperglycemia and represents one of the most significant global health challenges of the 21st century. Epidemiological data indicate that approximately 537 million adults aged 20–79 years were living with DM in 2021, with an estimated 6.7 million deaths attributed to the disease [1,2]. Among its subtypes, type 2 diabetes mellitus (T2DM) accounts for approximately 90% of all cases and is primarily associated with disturbances in carbohydrate and lipid metabolism. These metabolic disruptions result in insulin resistance (IR) and impaired insulin secretion due to pancreatic β-cell dysfunction [3].

The progression of T2DM is often preceded by an intermediate stage known as prediabetes, characterized by elevated blood glucose levels that remain below the diagnostic threshold for diabetes. According to the World Health Organization [4] and the American Diabetes Association [5], prediabetes is defined by fasting blood glucose levels between 6.1–6.9 mmol/L (110–125 mg/dL), impaired glucose tolerance in the two-hour oral glucose tolerance test (OGTT) with values between 7.8 and 11 mol/L (140–199 mg/dL), or glycated hemoglobin (HbA1c) levels ranging from 5.7 to 6.4%.

The transition from prediabetes to T2DM is strongly influenced by lifestyle factors, particularly dietary habits. The excessive consumption of sugar-sweetened beverages, often associated with to obesity, is a major contributor. Studies indicate that the daily intake of 250 mL of these beverages can result in an annual weight gain of 0.12 kg in adults and a body mass index (BMI) increase of 0.05 kg/m^2^ in children [6]. Over the past four decades, obesity—driven by an imbalance between energy intake and expenditure—has reached epidemic proportions worldwide [7].

Altered energy metabolism is a hallmark of both prediabetes and T2DM. In prediabetes, glucose intolerance primarily stems from IR in skeletal muscle and adipose tissue, leading to reduced glucose uptake in the postprandial state. Elevated fasting glucose levels are often associated with hepatic IR and increased gluconeogenesis [8]. As IR progresses, the dysregulation of glucose and fatty acid metabolism becomes more pronounced, characterized by impaired glucose uptake, enhanced glycolysis, and glycogenolysis, increased gluconeogenesis, and sustained lipogenesis—all of which are closely linked to alterations in key enzymatic pathways [9].

Mitochondria, the central organelles in cellular metabolism, play essential roles in oxidative phosphorylation, fatty acid metabolism, and the generation of reactive oxygen species (ROS). Mitochondrial function is regulated by three fundamental processes: biogenesis, dynamics, and bioenergetics. Mitochondrial biogenesis involves the coordinated synthesis of new mitochondria, including mitochondrial DNA (mtDNA) replication and the expression of nuclear-encoded proteins critical to mitochondrial function [10,11,12]. Mitochondrial dynamics refers to the continuous processes of fusion and fission that maintain organelle integrity, distribution, and adaptability. Mitochondrial bioenergetics relies on the electron transport chain (ETC) to generate ATP via oxidative phosphorylation through the transfer of electrons across complexes embedded in the inner mitochondrial membrane [10,11,12].

Mitochondrial dysfunction is a key driver of T2DM pathogenesis, contributing to energy imbalance by reducing ATP production, increasing ROS accumulation, and impairing ETC function [13]. Abnormalities in mitochondrial biogenesis and dynamics are strongly linked to the progression of IR [14]. Impaired mitochondrial function promotes metabolic inflexibility, reducing the cellular ability to switch between glucose and fatty acid substrates. Excessive mitochondrial fission and reduced fusion, commonly observed in diabetic conditions, decrease ATP generation and shift cells toward glycolysis. This metabolic shift leads to the accumulation of intermediates that disrupt insulin signaling pathways. Moreover, increased ROS generation further aggravates IR by inducing oxidative damage and promoting inflammation [15,16,17].

Melatonin (N-acetyl-5-methoxytryptamine) is a lipophilic indolamine synthesized from tryptophan, primarily by the pineal gland, though also produced in peripheral tissues [18]. While melatonin is well known for its role in regulating circadian rhythm, it exhibits pleiotropic biological effects, including anti-carcinogenic, immunomodulatory, and antioxidant properties, depending on the physiological context. Emerging evidence suggests that melatonin plays a significant role in metabolic modulation, influencing multiple pathways involved with the metabolic syndrome and DM [19,20,21,22]. In T2DM, melatonin modulates the hepatic energy metabolism by enhancing glycogenesis and suppressing gluconeogenesis, contributing to glycemic control. It also influences lipid metabolism by reducing total cholesterol, triglycerides, and LDL levels while elevating HDL concentrations [23,24,25,26,27,28]. Additionally, melatonin’s antioxidant properties counteract oxidative stress by scavenging ROS and enhancing the activity of endogenous antioxidant enzymes, thus protecting mitochondrial function in IR and T2DM [29,30,31]. Melatonin also regulates mitochondrial dynamics by inhibiting excessive fission and promoting fusion, stabilizing the ETC, and enhancing ATP production—mechanisms that support cellular energy homeostasis and mitigate metabolic disturbances linked to IR and T2DM [21].

Despite growing evidence supporting the beneficial effects of melatonin in DM, its role in the metabolic alterations associated with IR and prediabetes remains poorly understood. Further research is needed to elucidate its impact on energy metabolism, lipid homeostasis, OS, and mitochondria-associated events in the context of prediabetes. To this end, we investigated the effects of melatonin treatment in a Wistar rat model of prediabetes induced by a high-sugar diet combined with low-dose streptozotocin (STZ). This study aims to provide novel insights into the therapeutic potential of melatonin in alleviating metabolic dysfunction and mitochondrial impairments associated with prediabetes.

## 2. Results

### 2.1. Sucrose Solution Consumption Alters Daily Feed Intake in Prediabetic Wistar Rats

Weight gain remained comparable across all experimental groups throughout the study, with no significant differences observed in body mass index (BMI), Lee index, abdominal circumference, or body length. However, daily food intake (g/day) was significantly reduced in the pre-diabetes (PD) group compared to the control (C) group (*p* < 0.0001). Consequently, energy intake from chow was 43.96% lower in the PD group relative to control (*p* < 0.0001), but 16.63% higher in the PD-Mel group compared to PD (*p* = 0.0437). Energy intake from liquid consumption was elevated in both PD and PD-Mel groups, as the C group did not receive sucrose solution. Overall, total energy intake (kcal/day) in the PD group was 73.25% higher than in the control group (*p* < 0.0001), and melatonin treatment did not significantly alter this parameter. No differences in daily liquid intake were observed between groups (Table 1).

### 2.2. Melatonin Reduces Serum Glucose but Does Not Improve Glucose Intolerance

**Figure 1:**
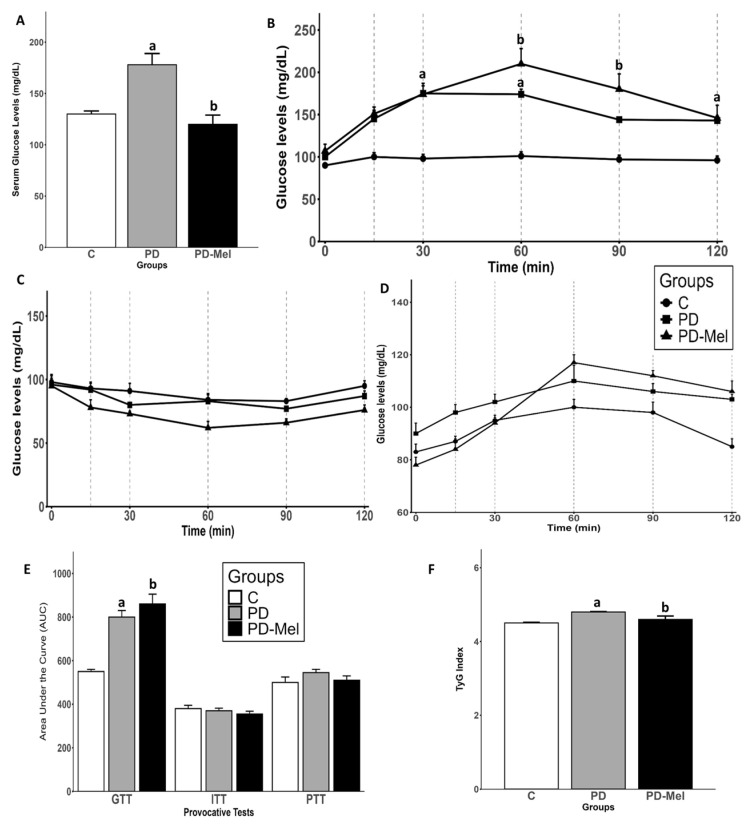
Glycemic parameters of rats from control, prediabetic, and prediabetic treated with melatonin groups. (**A**) Fasting serum glucose levels; (**B**) Intraperitoneal glucose tolerance test (GTT); (**C**) Insulin tolerance test (ITT); (**D**) Pyruvate tolerance test (PTT); (**E**) Area under curve (AUC) of previous provocative tests; and (**F**) TyG Index. Data are expressed as mean ± SEM and analyzed by one-way ANOVA followed by Tukey’s test for multiple comparisons with *p* value ≤ 0.05. Letters a: Differs statistically from C group; b: Differs statistically from PD group. C—control group (n = 10); PD—prediabetic group (n = 8); PD-Mel—prediabetic-melatonin-treated group (n = 9).

Fasting serum glucose was measured via cardiac puncture, while glucose tolerance was evaluated using glucose tolerance test (GTT), insulin tolerance test (ITT), and pyruvate tolerance test (PTT), via tail puncture. The combination of sucrose intake and STZ injection led to a 25.51% increase in serum glucose in the PD group compared to control (*p* = 0.0219). The melatonin treatment reduced serum glucose by 22.33% in the PD-Mel group (*p* = 0.0240) (Figure 1A). During the GTT, baseline glucose levels were similar across all groups. However, in PD groups, blood glucose remained elevated at 30 (*p* = 0.0052) and 60 min (*p* = 0.004). In the PD-Mel group, glucose levels increased significantly at 60 min (*p* < 0.0001) and remained high until 90 min (*p* = 0.0048) (Figure 1B). The area under the curve (AUC) analysis confirmed higher glucose intolerance in the PD group vs. control (*p* < 0.0001) and in the PD-Mel vs. PD (*p* = 0.0168) (Figure 1E). No significant differences were observed in ITT or PTT among groups (Figure 1C,D). The TyG index, an insulin resistance marker, was 6.03% higher in the PD group vs. control (*p* = 0.002), and melatonin reduced this index by 4.57% in the PD-Mel group (*p* = 0.0067) (Figure 1F).

### 2.3. Melatonin Reduces Fat Deposits and Influences Serum Lipid Profiles

Total fat content in the PD group was 109.79% higher than in the Control (*p* = 0.0002). Melatonin treatment the reduced fat content by 61.07% in the PD-Mel group (*p* < 0.0001), restoring levels to those of Control (Table 2). Retroperitoneal and epididymal fat increased by 191% (*p* < 0.0001) and 75.06% (*p* = 0.0068) in the PD group, respectively, and were both reduced by ~60% with melatonin (*p* < 0.0001).

The PD group exhibited dyslipidemia, with elevated triglyceride (TG) and total cholesterol (TC) levels compared to Control (*p* = 0.0008 and *p* = 0.0002, respectively). Melatonin normalized TG levels and reduced TC by 12.8% (*p* = 0.0065). (Table 2). Aspartate transaminase (AST) levels were approximately 30% lower in PD-Mel compared to PD group (*p* = 0.0226), with no changes in alanine transaminase (ALT) or lipoprotein levels.

Hepatic TG increased by 18.63% in PD vs. Control (*p* = 0.0049), and melatonin did not affect this parameter. Hepatic HDL was 4.95% lower in PD vs. Control (*p* < 0.0001), with no changes in hepatic TC or LDL levels (Table 2).

### 2.4. Melatonin Modulates the Expression of Genes Related to Energy Metabolism

**Figure 2:**
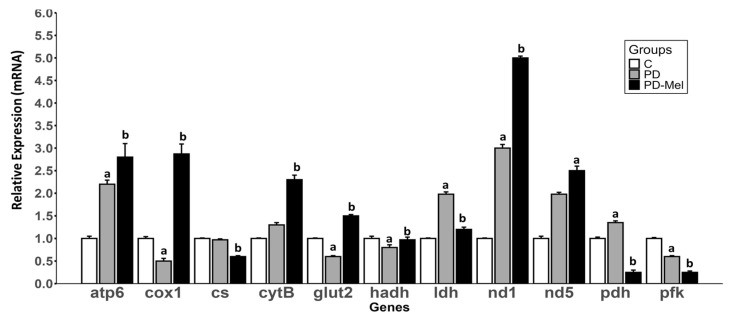
Gene expression panel of key enzymes related to hepatic energy metabolism of rats from Control, prediabetic, and prediabetic treated with melatonin groups. Differences were calculated using the ΔΔCt method, with β-actin used as the housekeeping gene. For comparative analysis, the control group was set as a calibrator (value = 1), and the relative expression levels in the other groups were calculated accordingly. Data expressed as mean ± SEM and analyzed by one-way ANOVA followed by Tukey’s test for multiple comparisons with *p* value ≤ 0.05. Letters a: Differs statistically from C group; b: Differs statistically from PD group. C—Control group. *Atp6*: ATP synthase subunit 6; *cox1*: cytochrome c oxidase 1; *cs*: citrate synthase, cytB: cytochrome b; *glut2*: glucose transporter type 2; *hadh*: hydroxyacyl-coA dehydrogenase; *ldh*: lactate dehydrogenase; nd1: NADH dehydrogenase subunit 1; *nd5*: NADH dehydrogenase subunit 5; *pdh*: pyruvate dehydrogenase subunit; *pfk*: phosphofrutokinase-1; C—control group (n = 10); PD—prediabetic group (n = 8); PD-Mel—prediabetic-melatonin-treated group (n = 9).

Gene expression analysis revealed significant changes in hepatic energy metabolism and ETC components (Figure 2). The ATP synthase subunit 6 (*Atp6*) was 139.02% higher in PD vs. Control (*p* = 0.0003), and 30.39% higher in PD-Mel vs. PD (*p* = 0.0408). Cytochrome c oxidase 1 (*Cox1;* ETC—complex IV) was reduced by 57.41% in the PD group (*p* = 0.0077), but increased by 607% in PD-Mel compared to PD (*p* < 0.0001). Citrate synthase (*Cs*) was downregulated in PD-Mel compared to PD (~39%, *p* < 0.0001). The expression of cytochrome b (*Cytb*), a subunit of ETC-complex III, was 73.84% higher in PD-Mel vs. PD (*p* < 0.0001).

In the PD group, glucose transporter type 2 (*Glut2*) expression was 45.1% lower in PD (*p* < 0.0001) and increased by 187.27% with melatonin (*p* < 0.0001). (Figure 2). Hydroxyacyl-CoA dehydrogenase (*Hadh*) expression was 24.15% higher in PD-Mel vs. PD (*p* = 0.0031), and the expression of lactate dehydrogenase (*Ldha*) increased by 99.7% in PD (*p* < 0.0001) and decreased by 45.98% after melatonin treatment (*p* < 0.0001).

Both the prediabetic state and melatonin treatment altered the mRNA expression of the components of the respiratory complex 1 (Figure 2). Expression of NADH dehydrogenase subunit 1 (*Nd1*) was 180% higher in PD vs. Control (*p* = 0.0462), and 110% higher in PD-Mel vs. PD (*p* = 0.0011). Pyruvate dehydrogenase subunit 1 (*Pdh1*) was 33.39% higher in PD group (*p* < 0.0001), but decreased by 80% with melatonin (*p* < 0.0001). Expression of phosphofrutokinase-1 liver-type (*Pfkl*) was lower in PD compared to Control (*p* < 0.0001), and lower in the PD-Mel compared to PD (*p* < 0.0001).

### 2.5. Melatonin Modulates the Activity of Enzymes Related to Energy Metabolism

**Figure 3:**
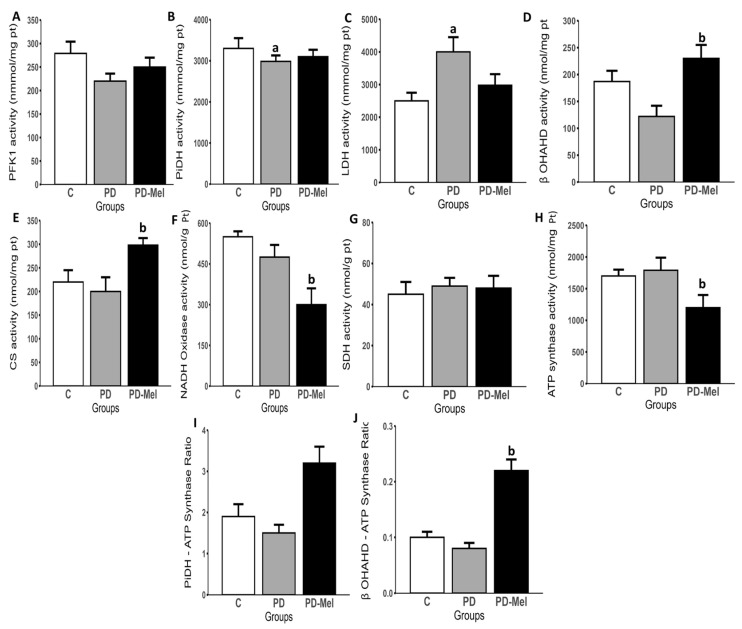
Activity of key enzymes of the hepatic energy metabolism of rats from Control, prediabetic, and prediabetic treated with melatonin groups. (**A**) PFK1 (Phosphofructokinase1); (**B**) PiDH (pyruvate dehydrogenase) complex; (**C**) LDH (lactate dehydrogenase); (**D**) β-OHADH (β-hydroxyacyl-coA dehydrogenase;) (**E**) CS (citrate synthase); (**F**) NADH oxidase; (**G**) SDH (succinate dehydrogenase); (**H**) ATP synthase; (**I**) Ratio between PiDH/ATP synthase activities; (**J**) Ratio between β-OHADH/ATP synthase activities. Data are expressed as the mean ± SEM and analyzed by one-way ANOVA followed by Tukey’s test for multiple comparisons with *p* value ≤ 0.05. Letters a: Differs statistically from group C; b: Differs statistically from PD group. C—control group (n = 10); PD—prediabetic group (n = 8); PD-Mel—prediabetic-melatonin-treated group (n = 9).

Activity analysis revealed significant alterations in key metabolic enzymes in prediabetic rats (Figure 3). Lactate dehydrogenase (LDH) activity was 62.67% higher in PD vs. control (*p* = 0.0380) (Figure 3C). β-hydroxyacyl-coA dehydrogenase (β-OHADH) activity increased by 79.45% in PD-Mel vs. PD (*p* = 0.0004). Citrate synthase (CS) activity was 22.21% higher in PD-Mel vs. Control (*p* = 0.0137), and 41.15% vs. PD (*p* = 0.0002) (Figure 3D,E). Both PD and PD-Mel groups exhibited an ~18% reduction in the pyruvate dehydrogenase complex (PiDH) activity (*p* < 0.0001 for both) (Figure 3B).

NADH oxidase activity was 36.44% lower in PD-Mel vs. PD (*p* = 0.0357), and ATP synthase activity decreased by ~35% (*p* = 0.0002) (Figure 3F,H). No significant changes were observed in phosphofructokinase (PFK1) or succinate dehydrogenase (SDH) activity (Figure 3A,G). To further explore the functional implications of these enzymatic changes, the ratios between PiDH/ATP synthase and β-OHADH/ATP synthase activities were analyzed. The β-OHADH/ATP synthase ratio was significantly higher in the PD-Mel group compared to the PD group (*p* < 0.0001) (Figure 3I,J).

### 2.6. Melatonin Alters the Expression of Mitochondrial Biogenesis and Dynamics Markers

**Figure 4:**
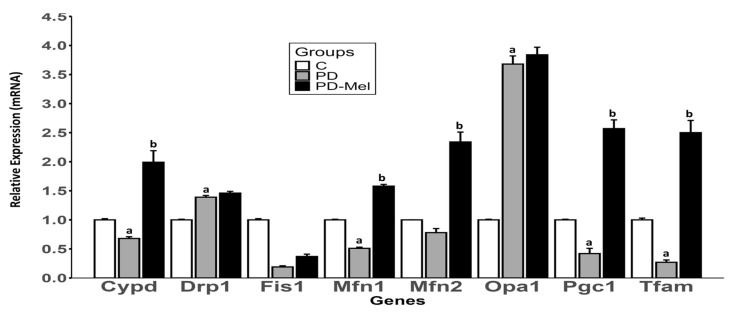
Gene expression panel of proteins related to hepatic mitochondrial events of rats from Control, prediabetic, and prediabetic treated with melatonin groups. Gene expression differences were calculated using the ΔΔCt method, with β-actin used as the housekeeping gene. For comparative analysis, the control group was set as a calibrator (value = 1), and the relative expression levels in the other groups were calculated accordingly. Data expressed as mean ± SEM and analyzed by one-way ANOVA followed by Tukey’s test with *p* value ≤ 0.05. Letters a: Differs statistically from C group; b: Differs statistically from PD group. Cypd: cyclophilin D; Drp1: dynamin-related protein; Fis1: mitochondrial fission protein 1; Mfn1: mitofusin-1; Mfn2: mitofusin-2; Opa: optic atrophy 1; Pgc1 peroxisome proliferator-activated receptor gamma coactivator; Tfam: mitochondrial transcription factor A. C—control group (n = 10); PD—prediabetic group (n = 8); PD-Mel—prediabetic-melatonin-treated group (n = 9).

Cyclophilin D (*Cypd*) expression was 31% lower in PD (*p* = 0.0023), and increased by 192% in PD-Mel (*p* < 0.0001) (Figure 4). Dynamin-related protein 1 (*Drp1*), a marker of mitochondrial fission, rose by ~40% in PD vs. Control (*p* < 0.0001). Mitofusin 1 (*Mfn1*) was 49% lower in PD vs. control (*p* < 0.0001), and increased by 209% with melatonin (*p* < 0.0001). The *Mfn2* expression also increased by 200% in PD-Mel (*p* < 0.0001).

Optic atrophy protein 1 (*Opa1*), a marker of mitochondrial fusion, had its expression increased by 267% in PD (*p* < 0.0001). Peroxisome proliferator-activated receptor gamma coactivator-1 alpha (*Pgc1-α*) was 57% lower in PD (*p* = 0.004) and increased by 502% in PD-Mel (*p* < 0.0001). Similarly, the mitochondrial transcription factor A (*Tfam*) expression was 73% lower in PD (*p* = 0.0024), and increased by 827% in PD-Mel (*p* < 0.0001) (Figure 4).

### 2.7. Melatonin Reduces Hepatic Oxidative Stress

**Figure 5:**
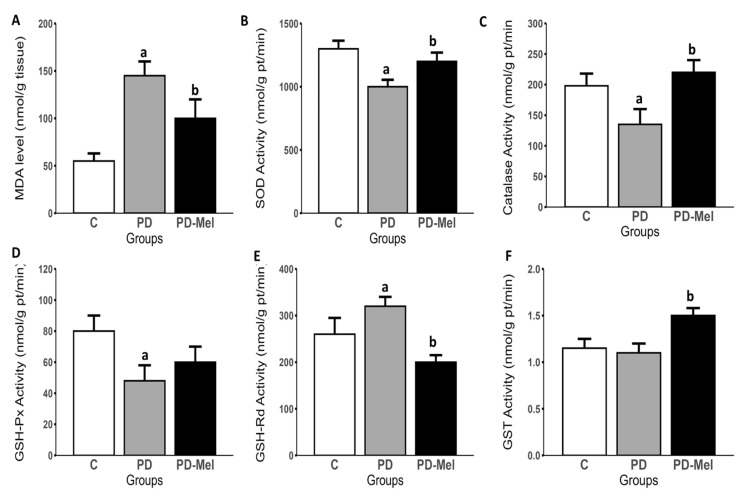
Hepatic oxidative stress parameters of rats from Control, prediabetic, and prediabetic treated with melatonin groups. (**A**) Malondialdehyde levels (MDA), and activity of (**B**) Superoxide dismutase (SOD); (**C**) Catalase; (**D**) Glutathione peroxidase (GSH-PX); (**E**) Glutathione reductase (GSH-Rd); (**F**) Glutathione transferase (GST). Data are expressed as mean ± SEM and analyzed by one-way ANOVA followed by Tukey’s test for multiple comparisons with *p* value ≤ 0.05. Letters a: Differs statistically from group C; b: Differs statistically from PD group. (C) control group (n = 10); PD—prediabetic group (n = 8); PD-Mel—prediabetic-melatonin-treated group (n = 9).

Malondialdehyde (MDA) levels increased by 135.44% in PD (*p* = 0.0002), and were reduced by 22.64% in PD-Mel (*p* = 0.0275) (Figure 5A). Superoxide dismutase (SOD) activity was reduced by 11% in PD (*p* < 0.0001) and increased by 16.42% in PD-Mel (*p* = 0.0018) (Figure 5B). Similarly, catalase (CAT) activity decreased by 29.39% in PD (*p* = 0.0246) and increased by 65.96% in PD-Mel (*p* = 0.0006) (Figure 5C).

Glutathione S-transferase (GST) activity increased significantly in PD-Mel (*p* = 0.0017) (Figure 5F). Glutathione reductase (GSH-Rd) activity rose by 25.10% in PD (*p* = 0.0001), and decreased by 35.67% in PD-Mel (*p* < 0.0001) (Figure 5E). Glutathione peroxidase (GSH-Px) activity decreased by 39.68% in PD (*p* = 0.0183) (Figure 5D). Reduced glutathione (GSH) levels dropped by ~37% in PD (*p* < 0.0001), and oxidized glutathione (GSSG) levels were 33.33% lower in PD vs. PD-Mel. OSI was elevated in PD and reduced with melatonin (Table 3).

### 2.8. Prediabetic Animals Exhibit Increased Collagen Deposition and Enlarged Hepatocyte Diameter

Hepatocyte diameter 122.97% higher in PD vs. C group (*p* = 0.0009). Melatonin treatment partially reversed this effect, reducing hepatocyte diameter by 22.82% (*p* = 0.0001). Collagen content increased by 351.61% in PD vs. control. Although melatonin reduced collagen by 22.85%, the difference was not statistically significant. Hepatocyte counts were unchanged among groups (Table 4).

## 3. Discussion

The findings of this study provide novel insights into the therapeutic potential of melatonin in mitigating metabolic dysfunction and mitochondrial abnormalities associated with prediabetes. Our results demonstrate that melatonin treatment significantly improves lipid and energy metabolism, reduces oxidative stress, and restores mitochondrial homeostasis in a rat model of prediabetes induced by chronic sucrose consumption and low-dose STZ administration. These effects were evident at both biochemical and molecular levels, highlighting melatonin’s pleiotropic actions in modulating key pathways involved in the pathophysiology of prediabetes.

High-sugar diets, particularly among overweight or obese individuals, are strongly associated with the development of T2DM. Sugar-sweetened beverages contribute significantly to daily caloric intake, leading to weight gain and metabolic disturbances [29]. The impact of liquid carbohydrate consumption on energy metabolism is well documented and contributes to a positive energy balance [30,31]. However, in our study, chronic sucrose consumption did not significantly alter body weight or BMI. Furthermore, the dietary pattern, i.e., sucrose and food consumption, remained unchanged throughout the entire experiment. Neither STZ administration nor melatonin treatment affected these parameters. These finding may be related to the study duration, as morphometric changes often require prolonged exposure to high-sucrose diets [19]. STZ may also have influenced weight regulation, given its known β-cell toxicity and associated catabolic effects [32].

Although sucrose solution may be more palatable than water, no differences in liquid consumption were observed—a recurring finding in our previous studies [20]. However, the presence of sucrose led to a compensatory decrease in food intake. This shift is critical as sucrose-supplemented water is energy dense, contributing to increased total caloric intake, even without increased liquid volume. This increased energy intake likely contributed to the fat accumulation, dyslipidemia, and insulin resistance observed in the PD group.

Despite the absence of weight gain, prediabetic animals exhibited increased fat deposition, evidencing the metabolic consequences of high-sucrose intake. Chronic sucrose consumption thus promoted adipose tissue expansion and dyslipidemia—both hallmark features of metabolic syndrome [33]. Importantly, melatonin treatment significantly attenuated these alterations, reducing fat deposits, lowering TC and TG levels, and increasing hepatic HDL. These results are consistent with previous reports using melatonin doses ranging from 2 to 50 mg/kg [27,28], including a study by Hadjzadeh et al. [26] employing 20 mg/kg, the same dose used herein. A limitation in this context is that we did not evaluate skeletal muscle mass or subcutaneous fat depots, which are important components of body composition. While adiposity was estimated using internal fat pads, more comprehensive and precise assessments—such as dual-energy X-ray absorptiometry (DEXA) in live animals—would provide better insights into total fat distribution and lean mass, thereby strengthening the interpretation of our findings.

The role of melatonin in regulating glycolytic homeostasis has been previously described [23,34,35]. Although melatonin did not improve glucose intolerance, it effectively normalized fasting serum glucose, which is typically disrupted due to hepatic IR [8]. The reduction in the TyG index, a robust predictor of IR, following melatonin treatment further supports an improvement in insulin sensitivity. Elevated ALT and AST levels are commonly recognized markers of hepatic damage and have been reported to increase following chronic sucrose consumption [19]. Interestingly, ALT and AST levels remained unchanged, suggesting no overt hepatic injury. These results suggest that metabolic dysfunction preceded hepatocellular damage, which is consistent with early-stage prediabetes.

The increased hepatic *Glut2* expression in melatonin-treated animals suggests improved glucose uptake. The observed downregulation of *Pfk1* expression, a gene regulated by insulin [36], may be associated with insulin deficiency. However, PFK1 enzymatic activity remained unchanged, possibly due to post-transcriptional regulatory mechanisms. Additionally, melatonin has been shown to modulate PXR, which downregulates *Glut2* and contributes to glucose intolerance [37,38], raising the possibility that melatonin’s effects are mediated in part through PXR inhibition.

Hepatic metabolic alterations are commonly reported in diabetic models under hypercaloric conditions [39]. Prediabetic animals showed reduced PiDH activity and increased *Ldha* expression, indicating a shift in pyruvate metabolism toward lactate production. Melatonin normalized *ldh* expression and LDH activity, a finding consistent with previous reports from our group [40]. This reduction in LDH activity also supports the safety of melatonin relative to biguanide-class drugs, e.g., metformin, which may increase lactate levels and pose a risk of lactic acidosis [41,42,43].

Melatonin also promoted a shift toward lipid oxidation. The upregulation of *Hadh* expression and increased β-OHADH activity in the PD-Mel group, alongside a higher β-OHADH/ATP synthase ratio, suggest that β-oxidation became the predominant ATP source. Under diabetic conditions, hepatic lipogenesis is often sustained, contributing to metabolic imbalances [39]. The increased CS activity may reflect a compensatory response to elevated lipid metabolism, supporting energy production while limiting lipotoxicity. Together with the reduced fat depots and improved lipid profiles, these results emphasize melatonin’s role in regulating lipid metabolism. Nevertheless, it is important to acknowledge certain methodological limitations. We did not assess state 3, mitochondrial respiration (i.e., ADP phosphorylation capacity), which could be evaluated using high-resolution respirometry to provide a more direct assessment of mitochondrial bioenergetic function. Additionally, ketone body levels were not measured, which would further clarify whether enhanced β-oxidation was accompanied by increased ketogenesis. These parameters should be considered in future studies to fully elucidate the metabolic consequences of melatonin treatment.

Mitochondria function is regulated by biogenesis, fusion, and fission processes, all of which are essential for cellular homeostasis [10]. To our knowledge, few prediabetes models assessed these parameters after melatonin’s treatment. Mitochondrial biogenesis genes have already been directly associated with the development of T2DM; decreased expression of *Pgc1-α* leads directly to decreased oxidative phosphorylation, lipid oxidation and thus contributes to increasing IR [44]. In this study, the downregulation of *Pgc1-α* and *Tfam* in the PD group suggests impaired mitochondrial biogenesis, consistent with metabolic disease models [15]. Thiazolidinediones (e.g., pioglitazone), which are known for upregulating PGC1-α, have shown similar metabolic benefits [45]. Melatonin treatment reversed these changes, upregulating *Pgc1-α* and *Tfam*, thereby promoting mitochondrial biogenesis and stability. In an in vitro model of sepsis-induced hepatocyte injury, melatonin treatment increased the protein levels of PGC-1α, NRF1, and TFAM, as well as reduced MDA levels and increased SOD activity [46].

In the T2DM scenario, mitochondrial dynamics are also affected by the imbalance in fission and fusion processes. Elevated *Drp1* expression in both PD and PD-Mel groups indicates persistent fission activity, a common feature in IR [16]. The persistent increase in *Drp1* expression even after melatonin treatment may indicate an incomplete restoration of mitochondrial homeostasis. However, melatonin markedly increased the expression of *Mfn1* and *Mfn2*, suggesting a partial recovery of fusion capacity. The significant downregulation of *Mfn1* in the PD group points to impaired fusion, contributing to mitochondrial fragmentation and dysfunction.

With high serum glucose levels, the Drp1/Mfn2 ratio increases, leading to mitochondrial fission and contributing to increased IR [47]. In our study, even with greater expression of Drp1 in the PD-Mel group, levels of *Mfn1 and Mfn2* were also elevated. *CypD*, a key regulator of the mitochondrial permeability transition pore (mPTP), plays a critical role in cell survival and apoptosis. The reduced *CypD* expression in PD animals suggests a shift toward a more closed mPTP state, possibly as an adaptive response to prevent excessive cell death under metabolic stress. *CypD* upregulation in the PD-Mel group may have restored mPTP homeostasis, potentially improving mitochondrial function by modulating permeability transition events. *Opa1*, another key fusion protein, was markedly upregulated in both PD and PD-Mel groups. *Opa1* is essential for maintaining mitochondrial cristae structure and function and alleviates ROS accumulation [48]. While excessive expression in the PD group may reflect a compensatory mechanism for impaired mitochondrial dynamics, the further increase in *Opa1* following melatonin treatment indicates that the indolamine enhances mitochondrial inner membrane remodeling, which could improve the ATP production efficiency and overall mitochondrial function.

Melatonin’s influence on mitochondrial metabolism was further reflected in the increased expression of ETC components and CS activity. However, these transcriptional changes did not translate into a corresponding increase in enzymatic activity, as evidenced by the reduction in complex I and V activities in the PD-Mel group. This may be attributed to excessive ROS production during enhanced β-oxidation, as the elevated NADH/NAD^+^ ratio can disrupt proton gradients and oxidative phosphorylation efficiency [49,50,51]. Complex I is particularly susceptible to ROS-mediated damage due to its limited electron-processing capacity, which can contribute to decreased ATP synthesis [52].

These previous findings are further supported by melatonin’s antioxidant effects, which occur through direct ROS scavenging or the indirect modulation of antioxidant enzymes [53]. The increased activity of SOD and CAT, coupled with reduced lipid peroxidation, supports this hypothesis. Elevated GST and reduced GSH-Rd activity may indicate a shift toward phase II detoxification and enhanced melatonin metabolism [53]. Despite a decline in GSH, GSH-Px activity remained low, possibly reflecting an overreliance on GSH for direct ROS neutralization. These findings are consistent with prior studies showing melatonin’s capacity to modulate oxidative stress in diabetic models [26,35,54,55]. Interestingly, while melatonin improved SOD and CAT activity, MDA and GSH may reflect more chronic oxidative damage or other non-enzymatic pathways.

Liver damage in metabolic disorders can progress from steatosis to fibrosis and, eventually, cirrhosis. Studies have demonstrated melatonin’s protective role in diabetic conditions by mitigating hydropic degeneration, pro-necrotic lesions [53], and fibrosis, including a reduction in collagen accumulation [56]. Our histological analysis revealed increased collagen deposition and hepatocyte hypertrophy in prediabetic animals, consistent with early fibrotic remodeling [20]. While melatonin treatment did not significantly reduce collagen accumulation, it attenuated hepatocyte hypertrophy, a marker of hepatic dysfunction. These data suggest that melatonin exerts hepatoprotective effects primarily through modulation of cellular hypertrophy rather than fibrotic resolution. This aligns with previous studies showing melatonin’s ability to modulate lipid and energy metabolism in the liver [23,24,27,28].

**Figure 6:**
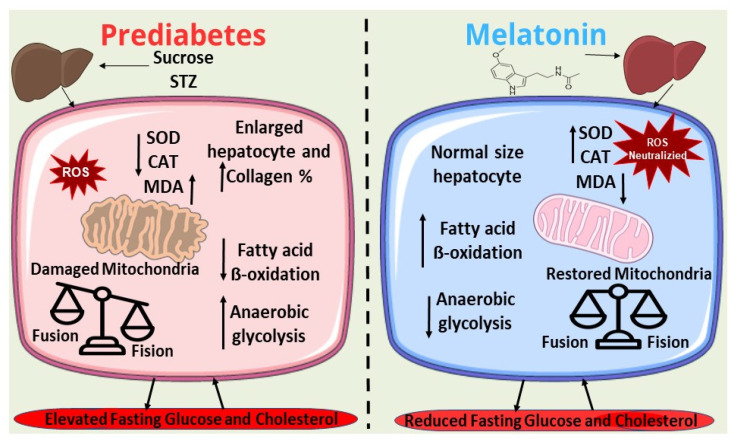
Serum and hepatic alterations caused by sucrose and streptozotocin (STZ) exposition and possible mechanisms by which melatonin treatment improves prediabetic condition.

In this study, melatonin treatment improved key biochemical markers, including TC and TG levels, decreased fat deposits, and increased hepatic HDL levels. Additionally, melatonin enhanced β-oxidation, a key process in lipid metabolism likely contributing to the observed modulation of lipid parameters. While melatonin did not improve ETC function directly, possibly due to the persistent OS from fatty acid oxidation byproducts, it significantly reduced anaerobic glucose oxidation, an important finding given that elevated lactate levels are a common side effect of antidiabetic drugs such as metformin. For the first time, we demonstrated that melatonin treatment impacts mitochondrial events which are involved in the prediabetes stage (Figure 6).

### Study Limitations

Despite the promising results, this study has additional limitations. While we assessed hepatic metabolic flux through enzyme expression and activity assays, advanced techniques such as isolated hepatocyte studies or ex vivo liver perfusion could provide a more comprehensive evaluation of metabolic pathways. Western blot analysis was also not conducted to validate protein expression levels, limiting the depth of our molecular insights.

## 4. Materials and Methods

### 4.1. Animals and Experimental Protocol

This study was conducted in compliance with the guidelines of the National Council for the Control of Animal Experimentation (CONCEA), Brazil, and was approved by the Ethics Committee for the Use of Animals (CEUA) of the State University of Northern Paraná (UENP), Brazil (protocol number: 001/2023). The experimental model was initially designed to induce T2DM in Wistar rats, adapting the protocol described by Salido and collaborators [57], by combining chronic sucrose consumption with a single injection of STZ. According to established criteria, T2DM in rats is confirmed when blood glucose levels exceed 200 mg/dL [58]. While this threshold was not reached in our study, the observed hyperglycemia and glucose intolerance were consistent with a prediabetic state, as characterized by Barrière et al. [59].

Thirty male Wistar rats (30 days old) were obtained from the central animal facility of the State University of Londrina, Brazil and housed individually under controlled conditions (temperature: 22 ± 3 °C, light/dark cycle: 12 h, humidity: 60 ± 5%). Following an acclimatization period, the animals were randomly assigned into two experimental groups. Both groups received ad libitum access to a standard rodent chow (Nuvilab-CR1, Nuvital, Colombo, Brazil); however, the control group (C; n = 10) was provided with autoclaved, filtered water, while the prediabetic group (PD; n = 20) received a 40% sucrose solution as the sole drinking source.

After four weeks, the PD group was further subdivided into two groups: untreated prediabetic rats (PD; n = 10) and prediabetic rats receiving melatonin (PD-Mel; n = 10). To induce prediabetes, both subgroups received a single intraperitoneal injection of STZ (50 mg/kg) dissolved in ice-cold 100 mM citrate buffer (pH 4.5). Seven days after STZ administration, blood glucose levels were assessed to confirm the prediabetic condition. Subsequently, the PD-Mel group was treated with melatonin (25 mg/kg), administered intraperitoneally three times per week for four weeks. Melatonin was prepared in a vehicle solution containing 99.5% ethanol and diluted in 0.9% NaCl. The absence of a melatonin-only control group is justified on ethical, scientific, and practical grounds. To stay in few rationalities: by not including this group, the study remains focused on the primary objective of understanding the effects of prediabetes in the presence of melatonin treatment, and by following the 3Rs, treating a control group with melatonin when melatonin’s effect on healthy animals is already well established could expose the animals to unnecessary treatments.

The dose of melatonin was selected based on previous studies demonstrating therapeutic properties in T2DM models using dose ranging from 0.2 to 50 mg/kg [54]. We adopted an intermediate dose of 25 mg/kg to reflect a balance between safety and efficacy. The chosen dose is consistent with studies using similar models and dosing regimens [26,27], although different routes of administration were used. Notably, during the experimental period, three animals died following STZ administration.

### 4.2. Nutritional and Morphometric Parameters

Throughout the study, body weight, food consumption, and water intake were recorded weekly in the morning. Food and liquid consumption were determined by calculating the difference between the total amount provided and the residual quantity in each cage. These data, along with their respective caloric values (3.09 kcal/g and 1.6 kcal/mL) [19], were used to calculate the energy derived from chow, from the sucrose solution, and the total energy intake. At the end of the experimental period, animals were anesthetized, and body morphometric parameters—including body weight and total body length—were measured to calculate the Body Mass Index (BMI: g/cm^2^ = body weight/length^2^) and the Lee index ((g/cm) = cube root of body weight/naso-anal length). The Lee index, similarly to BMI, is a quick, and non-invasive method to estimate adiposity in rats, which correlates with total body fat mass and is often reflective of metabolic alterations [60,61].

### 4.3. Intraperitoneal Glucose, Insulin, and Pyruvate Tolerance Tests

In the final week of the experiment, metabolic tolerance tests were conducted to evaluate glucose metabolism. The Glucose Tolerance Test (GTT), Insulin Tolerance Test (ITT), and Pyruvate Tolerance Test (PTT) were performed eight, five, and two days before euthanasia, respectively. For the GTT and ITT, animals underwent a 8 h fasting period. The GTT was conducted by administering a 20% sucrose solution (2 g/kg, i.p.), followed by blood glucose measurements at 0, 15, 30, 60, 90, and 120 min. For the ITT, animals received an intraperitoneal injection of human insulin (1 U/kg, HumulinTM Eli Lilly, São Paulo, Brazil), with blood glucose levels monitored at the same time intervals. The PTT was performed after 16 h of fasting to evaluate gluconeogenesis. Rats were administered an intraperitoneal injection of a 25% pyruvate solution (2 g/kg), and blood glucose levels were measured at 0, 15, 30, 60, 90, and 120 min. All blood glucose readings were obtained using a digital glucometer (On Call^®^ Plus II, ACON Biotech, Hangzhou, China).

### 4.4. Collection of Biological Material

At the end of the experimental period, animals fasted for 6 h were anesthetized with ketamine (90 mg/kg) and xylazine (10 mg/kg) and euthanized via cardiac puncture. Blood samples were centrifuged (RCF 85.75× *g*, 10 min), and serum aliquots were stored at −80 °C for biochemical analyses, including alanine transaminase (ALT), aspartate aminotransferase (AST), glucose, triacylglycerol (TG), urea, creatinine, total cholesterol (TC), and high-density lipoprotein (HDL) levels. All biochemical measurements were performed using commercial kits (Gold Analisa^®^) and read in a microplate reader (MultiSkan Skyhigh, Thermo Fisher Scientific Inc., MA, USA). The Friedewald equation was used to estimate low-density lipoprotein (LDL) levels: [LDL] = (TC − HDL) − (TG/5). The Triglyceride–Glucose (TyG) Index was calculated as an indirect marker of insulin resistance using the equation: Tyg = ln (fasting TG × fasting glucose/2).

Following euthanasia, white adipose tissue deposits, epididymal, visceral, and retroperitoneal, were excised and weighed. Liver samples were collected, weighed, and immediately stored at −80 °C for subsequent oxidative stress assessments and metabolic analyses. The same biochemical determinations performed on serum were also conducted on hepatic tissue.

### 4.5. Total RNA Extraction and cDNA Synthesis

Liver samples were homogenized in TRIzol^®^ reagent and stored at −80 °C until RNA extraction. Total RNA was isolated using the TRIzol^®^/Chloroform/Isopropanol method, following the manufacturer’s instructions. RNA purity and concentration were assessed using a NanoDrop 2000 spectrophotometer (Thermo Fisher Scientific, Waltham, MA, USA), with quality thresholds set at OD 260/280 ≥ 1.8 and OD 260/230 ≥ 1.0. Complementary DNA (cDNA) synthesis was performed using the SuperScript First Strand Supermix kit (Invitrogen, Carlsbad, CA, USA), following the manufacturer’s protocol. Random primers were used to ensure the comprehensive reverse transcription of RNA into cDNA.

### 4.6. Gene Expression Analysis

Quantitative PCR (qPCR) was employed to assess gene expression profiles using the SYBR Green Master Mix (qPCR^®^, Promega, Madison, WI, USA). Each reaction was performed in triplicate, with gene-specific primers (Table 5). 2^−ΔΔCt^ method was employed to quantify relative gene expression. β-ACTIN was selected as the internal reference gene for normalization. Fold changes and standard deviations were determined from three independent experiments, each conducted in duplicate.

### 4.7. Hepatic Energy Metabolism

To evaluate the hepatic energy metabolism, 200 mg of liver tissue was homogenized and subjected to centrifugation (RCF 10,360× *g*, 10 min). The resulting supernatant was collected, while the pellet was resuspended in 0.1 mol/L sodium phosphate buffer containing 250 mmol/L sucrose and 2 mmol/L EDTA for further enzymatic analyses. The activity of the enzymes NADH-oxidase and ATP synthase was analyzed using the cell precipitate and the supernatant was used for the other analyses. The enzymatic activity of key metabolic markers was assessed, including phosphofructokinase (PFK; E.C.2.7.1.11.), citrate synthase (CS; E.C.4.1.3.7.), lactate dehydrogenase (LDH; E.C.1.1.1.27.), β-hydroxyacyl-coenzyme A dehydrogenase (β-OHADH; E.C.1.1.1.35.), pyruvate dehydrogenase (PiDH; E.C.1.2.1.51.), succinate dehydrogenase (SDH; E.C.1.3.99.1.), NADH-oxidase (E.C.1.6.99.5.), and ATP synthase (E.C.7.1.2.2).

The reaction medium for each enzyme was prepared as follows:

− PFK: 50 mmol/L Tris-HCl buffer (pH 8.0), 10 mmol/L MgCl_2_, 1U glyceraldehyde-3-phosphate dehydrogenase, 1 U aldolase, 2.5 U triose phosphate isomerase, 0.12 mmol/L NADH, 0.75 mmol/L ATP, and 6 mmol/L fructose-6-phosphate.
− CS: 50 mmol/L Tris-HCl buffer (pH 8.0), 0.3 mmol/L acetyl-CoA, 0.1 mmol/L 5,5'-dithiobis-(2-nitrobenzoic acid) (DTNB), and 0.5 mmol/L oxaloacetate.
− LDH: 50 mmol/L Tris-HCl buffer (pH 7.4), 0.14 mmol/L NADH, and 1 mmol/L sodium pyruvate.
− β-OHADH: 0.1 mol/L Tris-HCl buffer (pH 7.0), 5 mmol/L EDTA, 0.05 mmol/L acetoacetyl-coenzyme A, and 0.1 mmol/L NADH.
− PiDH: 2.5 mmol/L NAD, 0.1 mmol/L coenzyme A, 0.2 mmol/L thiamine pyrophosphate, 0.3 mmol/L dithiothreitol (DTT), 1 mmol/L MgCl_2_, 5 mmol/L pyruvate, 0.08 mmol/L nitrotetrazolium blue (NBT), and 1 mg bovine serum albumin (BSA), with 3 mmol/L sodium pyruvate and 0.05 mmol/L phenazine metasulfate in 50 mmol/L potassium phosphate buffer (pH 7.4).
− SDH: 50 mmol/L potassium phosphate buffer (pH 7.4), 10 mmol/L sodium succinate, 0.36 mmol/L phenazine metasulfate, and 0.12 mmol/L dichlorophenolindophenol (DPIP).
− NADH-oxidase: 80 mmol/L sodium phosphate buffer (pH 7.4), 50 mmol/L EDTA, and 0.2 mmol/L NADH.
− ATP synthase: 0.11 mmol/L MgCl_2_, 0.02 mmol/L NADH, 0.25 mmol/L phosphoenolpyruvate (PEP), 0.2 mmol/L ATP, 1686 U LDH, and 4001.18 U pyruvate kinase.

Enzymatic activity was determined at 25 °C using a microplate reader (μQuant-MQX 200) controlled via Kcjunior software (Bio-Tec Instruments, Winooski, VT, USA).

### 4.8. Hepatic Oxidative Stress

To assess oxidative stress markers, 200 mg of liver tissue was homogenized in 5 mL of ice-cold 0.1 mol/L sodium phosphate buffer (pH 7.4) using a Potter–Elvehjem homogenizer on ice. The homogenate was centrifuged (RCF 10,360× *g*, 10 min), and the supernatant was used for biochemical analyses. Markers of oxidative stress included total protein quantification [62], lipid peroxidation (malondialdehyde levels), total glutathione, reduced glutathione (GSH), and oxidized glutathione [63], and the activities of key antioxidant enzymes, such as glutathione peroxidase (GSH-Px; E.C.1.11.1.9.) [64], glutathione-S-transferase (GST; E.C.2.5.1.18.) [65], superoxide dismutase (SOD; E.C.1.15.1.1.) [66], and catalase (CAT; E.C.1.11.1.6.) [67]. All enzymatic assays were conducted at 25 °C using a microplate reader (μQuant-MQX 200) with Kcjunior software (Bio-Tec Instruments, Winooski, VT, USA).

### 4.9. Histopathological Analysis

For histopathological evaluation, liver samples were fixed in Bouin’s solution for 24 h, dehydrated, cleared, and embedded in paraffin. Semi-serial sections (5 µm thick) were obtained and stained for microscopic analysis.

Liver architecture was examined using a Zeiss AxioLab 5 light microscope (Zeiss, Oberkochen, Germany) equipped with a high-resolution digital camera. Thirty images per animal were captured at 200× magnification, focusing on the peri-central vein region. Hematoxylin and eosin (H&E) staining was used to determine hepatocyte density and morphometry (100 hepatocytes/animal) within a fixed field area (0.216 mm^2^). Image analysis was performed using ImageProPlus 4.5 software (Media Cybernetics, Rockville, MD, USA). To assess fibrosis, additional sections were stained with PicroSirius Red to detect type I and III collagen deposition. Quantitative collagen analysis was performed using ImageJ FIJI 1.54i software, applying RGB stack segmentation with the same parameters used for H&E-stained sections.

### 4.10. Statistical Analysis

Data were analyzed using one-way analysis of variance (ANOVA), followed by Tukey’s post-hoc test. Results are expressed as mean ± standard error of mean (SEM). Statistical significance was considered at *p* < 0.05. All analyses were conducted using GraphPad Prism 8.0.2 (GraphPad Software, San Diego, CA, USA).

## 5. Conclusions

In this study, melatonin treatment partially restored glycolytic homeostasis in prediabetic rats by lowering fasting glucose levels and reducing reliance on anaerobic glycolysis, as evidenced by decreased LDH expression and activity. The treatment also improved lipid metabolism, as demonstrated by reduced fat accumulation, lower serum triglyceride and total cholesterol levels, and increased hepatic HDL content. These findings indicate that melatonin promotes a favorable shift in both glucose and lipid handling in the liver.

In parallel, melatonin enhanced the activity of key antioxidant enzymes, including SOD and CAT, contributing to reduced hepatic oxidative stress. Importantly, our data provide compelling evidence of mitochondrial dysfunction in the prediabetic state and demonstrate that melatonin modulates the expression of genes involved in mitochondrial biogenesis, integrity, fusion, and fission. Taken together, these results highlight the potential of melatonin as a therapeutic agent to counteract metabolic and mitochondrial disturbances associated with prediabetes.

## Figures and Tables

**Table 1:** Morphometric and nutritional parameters of rats from control, prediabetic, and prediabetic treated with melatonin groups.

Parameters	Groups		
	C	PD	PD-Mel
Body weight gain (g)	212.3 ± 18.27	244.4 ± 20.39	197.7 ± 76.36
BMI (g/cm^2^)	0.63 ± 0.02	0.63 ± 0.01	0.55 ± 0.04
Lee index (g/cm)	11.71 ± 0.22	11.85 ± 0.37	11.08 ± 0.76
Abdominal circumference (cm)	16.1 ± 1.37	16.00 ± 0.81	15.22 ± 1.3
Body length (cm)	24.5 ± 0.53	24.38 ± 0.74	25.13 ± 1.8
Food consumption (g/day)	27.87 ± 0.47	15.51 ± 0.5 ^a^	16.85 ± 1.18
Liquid consumption (mL/day)	52.7 ± 1.47	50.14 ± 2.4	65 ± 7.57
Energy intake from food (kcal/day)	83.98 ± 1.45	47.06 ± 2.27 ^a^	50.89 ± 2.55
Energy intake from liquid (kcal/day)	0 ± 0.00	87.53 ± 4.23 ^a^	95.6 ± 3.77
Total energy intake (kcal/day)	83.98 ± 1.45	145.5 ± 8.73 ^a^	149.1 ± 6.64

Data expressed as mean ± SEM and analyzed by one-way ANOVA followed by Tukey’s test for multiple comparisons. ^a^ Differs statistically from C group. C—control group (n = 10); PD—prediabetic group (n = 8); PD-Mel—prediabetic-melatonin-treated group (n = 9).

**Table 2:** Fat deposits, serum and liver biochemical parameters of rats from control, prediabetic, and prediabetic treated with melatonin groups.

Parameters	Groups		
	C	PD	PD-Mel
Epididymal fat deposit (mg)	3.28 ± 0.22	5.75 ± 0.38 ^a^	2.08 ± 0.49 ^b^
Visceral fat deposit (mg)	2.33 ± 0.18	4.14 ± 0.34	2.31 ± 0.57
Retroperitoneal fat deposit (mg)	2.24 ± 0.31	6.54 ± 0.57 ^a^	2.48 ± 0.81 ^b^
Sum of fat depots (mg)	7.84 ± 1.85	16.45 ± 3 ^a^	6.4 ± 4.89 ^b^
Serum triglycerides levels (mg/dL)	101 ± 9.50	143 ± 29.31 ^a^	118 ± 34.11
Serum cholesterol levels (mg/dL)	105 ± 2.8	128 ± 4.51 ^a^	112 ± 2.41 ^b^
Serum HDL levels (mg/dL)	55 ± 2.81	61 ± 3.49	50 ± 5.51
Serum LDL levels (mg/dL)	28 ± 4.17	36 ± 3.35	27 ± 3.00
Serum ALT levels (U/L)	29 ± 2.07	20 ± 2.44	24 ± 4.35
Serum AST levels (U/L	43 ± 2.42	44 ± 2.74	30 ± 4.81 ^b^
Liver triglycerides levels (mg/dL)	87 ± 2.28	103 ± 4.02 ^a^	100 ± 3.10
Liver cholesterol levels (mg/dL)	94 ± 0.84	97 ± 1.17	95 ± 0.76
Liver HDL levels (mg/dL)	21 ± 0.17	20 ± 0.12 ^a^	20 ± 0.11
Liver LDL levels (mg/dL)	57 ± 7.43	55 ± 5.34	53 ± 2.22

Data expressed as mean ± SEM and analyzed by one-way ANOVA followed by Tukey’s test for multiple comparisons with *p* value ≤ 0.05. Letters ^a^: Differs statistically from C group; ^b^: Differs statistically from PD group. C—control group (n = 10); PD—prediabetic group (n = 8); PD-Mel—prediabetic-melatonin-treated group (n = 9).

**Table 3:** Liver glutathione pool and OSI of rats from the Control, prediabetic and prediabetic treated with melatonin groups.

Parameters	Groups		
	C	PD	PD-Mel
Total Glutathione (nmol/mg tissue)	2.97 ± 0.10	2.21 ± 0.15	1.93 ± 0.29
GSH (nmol/mg tissue)	2.11 ± 0.08	1.31 ± 0.07 ^a^	1.33 ± 0.15
GSSG (nmol/mg tissue)	0.52 ± 0.04	0.45 ± 0.02	0.3 ± 0.04 ^b^
OSI (%)	10.55 ± 0.72	14.47 ± 1.75 ^a^	10.34 ± 1.56 ^b^

Data expressed as mean ± SEM and analyzed by one-way ANOVA followed by Tukey’s test for multiple comparisons. Letters ^a^: Differs statistically from C group; ^b^: Differs statistically from PD group. GSH—reduced glutathione; GSSG—oxidized glutathione; OSI—oxidative stress index. C—control group (n = 10); PD—prediabetic group (n = 8); PD-Mel—prediabetic-melatonin-treated group (n = 9).

**Table 4:** Histopathological parameters of rats from the Control, prediabetic, and prediabetic treated with melatonin groups.

Parameters	Groups			
	C	PD	PD-Mel	
Hepatocyte number	237.3 ± 6.86	221.6 ± 8.99	232.6 ± 10.85	
Hepatocyte morphometry (µm)	2.96 ± 0.07	3.64 ± 0.09 ^a^	2.82 ± 0.10 ^b^	
Collagen (%)	0.31 ± 0.02	1.40 ± 0.19 ^a^	1.08 ± 0.14	

Data expressed as mean ± SEM and analyzed by one-way ANOVA followed by Tukey’s test for multiple comparisons. Percentage of collagen refers to the proportion of the stained area in liver histological sections. Letters ^a^: Differs statistically from C group; ^b^: Differs statistically from PD group. C—control group (n = 10); PD—prediabetic group (n = 8); PD-Mel—prediabetic-melatonin-treated group (n = 9).

**Table 5:** Sequence of primers used for gene expressions.

Gene (ID)	Primer	5’–3’ Sequence	Cycle Condition	Product Size (bp)
ACTB (81822)	Forward	CCTCTATGCCAACACAGTGC	95 °C—8 s; 61.5 °C—8 s; 72 °C—8 s	206
	Reverse	CCTGCTTGCTGATCCACATC		
Slc2a2 (25351)	Forward	TGTGAAAGTCATGCATGTGGC	95 °C—8 s; 61.5 °C—8 s; 72 °C—8 s	104
	Reverse	AGGCCAGGGATTGGTGTTAAA		
Hadh (113965)	Forward	AGTCTGGACTTGACCTTCTTGG	95 °C—8 s; 61.5 °C—8 s; 72 °C—8 s	138
	Reverse	TCTGAGGGCCCATTTTGATGT		
Pdh1 (29554)	Forward	TCCCCAGCTTGGCTTATTGT	95 °C—8 s; 61.5 °C—8 s; 72 °C—8 s	132
	Reverse	GGGTGGCTTCAAGTTTGCTTT		
Pfkl (25741)	Forward	AACAATGTCCCTGGCACTGA	95 °C—8 s; 61.5 °C—8 s; 72 °C—8 s	189
	Reverse	ACTCTCCATTGCAGCGTTGA		
Cs (170587)	Forward	ACCATGACGGTGGCAATGTA	95 °C—8 s; 61.5 °C—8 s; 72 °C—8 s	210
	Reverse	TGGTTTGCTAGTCCATGCAGA		
Ldha (24533)	Forward	TCCTCAGCGTCCCATGTATC	95 °C—8 s; 61.5 °C—8 s; 72 °C—8 s	193
	Reverse	TCCATAGAAACCCTGCTGCA		
Tfam (21780)	Forward	TCCACAGAACAGCTACCCAA	95 °C—8 s; 66 °C—8 s; 72 °C—8 s	84
	Reverse	CCACAGGGCTGCAATTTTCC		
Cypd (105675)	Forward	CCCTCCTGGTCACTGTGAAT	95 °C—8 s; 66 °C—8 s; 72 °C—8 s	186
	Reverse	GCCAGAAGACACTTCCCTCT		
Nd1 (17716)	Forward	ATTACTTCTGCCAGCCTGACC	95 °C—8 s; 66 °C—8 s; 72 °C—8 s	70
	Reverse	GGCCCGGTTTGTTTCTGCTA		
Nd5 (4540)	Forward	CCTGGCACTGAGTCACCATA	95 °C—8 s; 66 °C—8 s; 72 °C—8 s	214
	Reverse	TTGTTGGCTGAGGTGAGGAT		
Atp6 (100503946)	Forward	TCCCAATCGTTGTAGCCATCA	95 °C—8 s; 66 °C—8 s; 72 °C—8 s	76
	Reverse	AGACGGTTGTTGATTAGGCGT		
Cox1 (17708)	Forward	ATCACTACCAGTGCTAGCCG	95 °C—8 s; 66 °C—8 s; 72 °C—8 s	84
	Reverse	CCTCCAGCGGGATCAAAGAA		
Drp1 (74006)	Forward	GTCGAGTCCCCATTCATTGC	95 °C—8 s; 66 °C—8 s; 72 °C—8 s	151
	Reverse	ACTACGACGATCTGAGGCAG		
Mfn1 (67414)	Forward	GCAGACAGCACATGGAGAGA	95 °C—8 s; 61 °C—8 s; 72 °C—8 s	83
	Reverse	GATCCGATTCCGAGCTTCCG		
Mfn2 (170731)	Forward	TGCACCGCCATATAGAGGAAG	95 °C—8 s; 61 °C—8 s; 72 °C—8 s	78
	Reverse	TCTGCAGTGAACTGGCAATG		
Opa1 (74143)	Forward	ACCTTGCCAGTTTAGCTCCC	95 °C—8 s; 61 °C—8 s; 72 °C—8 s	82
	Reverse	TTGGGACCTGCAGTGAAGAA		
Fis1 (66437)	Forward	CAAAGAGGAACAGCGGGACT	95 °C—8 s; 61 °C—8 s; 72 °C—8 s	95
	Reverse	ACAGCCCTCGCACATACTTT		
Cytb (17711)	Forward	GGCTACGTCCTTCCATGAGG	95 °C—8 s; 61 °C—8 s; 72 °C—8 s	75
	Reverse	TGGGATGGCTGATAGGAGGT		

ACTB: β-actin; Slc2a2: coding gene for GLUT2; Hadh: coding gene for hydroxyacyl-CoA dehydrogenase; Pdh1: coding gene for alpha 1 subunit of pyruvate dehydrogenase complex; Pfkl: coding gene for phosphofrutokinase-1 liver-type; Cs: coding gene for citrate synthase; Ldha: coding gene for lactate dehydrogenase A; Tfam: mitochondrial transcription factor A; Cypd: cyclophilin D; Nd1: NADH dehydrogenase subunit 1; Nd5: NADH dehydrogenase subunit 5; Atp6: ATP synthase subunit 6; Cox1: cytochrome c oxydase 1; Drp1: dynamin-related protein 1; Mfn1: mitofusin-1; Mfn2; mitofusin-2; Opa1: optic atrophy 1; Fis1: mitochondrial fission protein 1; Cytb: cytochrome b.

## Data Availability

Data is contained within the article.
